# Multi-millijoule few-cycle mid-infrared pulses through nonlinear self-compression in bulk

**DOI:** 10.1038/ncomms12877

**Published:** 2016-09-13

**Authors:** V. Shumakova, P. Malevich, S. Ališauskas, A. Voronin, A. M. Zheltikov, D. Faccio, D. Kartashov, A. Baltuška, A. Pugžlys

**Affiliations:** 1 Photonics Institute, TU Wien, Gusshausstrasse 27-387, A-1040 Vienna, Austria; 2 Physics Department, International Laser Center, M.V. Lomonosov Moscow State University, 119992 Moscow, Russia; 3 Russian Quantum Center, ul. Novaya 100, Skolkovo, Moscow region 143025, Russia; 4 Department of Physics and Astronomy, Texas A&M University, College Station, 77843-4242 Texas, USA; 5 Institute of Photonics and Quantum Sciences, David Brewster Building, DB1.12 Heriot-Watt University Edinburgh, Edinburgh, EH14 4AS UK; 6 Institute for Optics and Quantum Electronics, Friedrich-Schiller University Jena, Max-Wien Platz 1, 07743 Jena, Germany; 7 Center for Physical Sciences & Technology, Savanoriu Ave. 231, LT-02300 Vilnius, Lithuania

## Abstract

The physics of strong-field applications requires driver laser pulses that are both energetic and extremely short. Whereas optical amplifiers, laser and parametric, boost the energy, their gain bandwidth restricts the attainable pulse duration, requiring additional nonlinear spectral broadening to enable few or even single cycle compression and a corresponding peak power increase. Here we demonstrate, in the mid-infrared wavelength range that is important for scaling the ponderomotive energy in strong-field interactions, a simple energy-efficient and scalable soliton-like pulse compression in a mm-long yttrium aluminium garnet crystal with no additional dispersion management. Sub-three-cycle pulses with >0.44 TW peak power are compressed and extracted before the onset of modulation instability and multiple filamentation as a result of a favourable interplay between strong anomalous dispersion and optical nonlinearity around the wavelength of 3.9 μm. As a manifestation of the increased peak power, we show the evidence of mid-infrared pulse filamentation in atmospheric air.

## Abstract (web summary)

Exploring strong-field laser interaction requires pulses that are both energetic and short. Here, the authors demonstrate a mid-IR soliton-like pulse compression in a mm-long YAG crystal, reaching the multi-millijoule energy range and showing pulse filamentation in atmospheric air.

## Graphical abstract

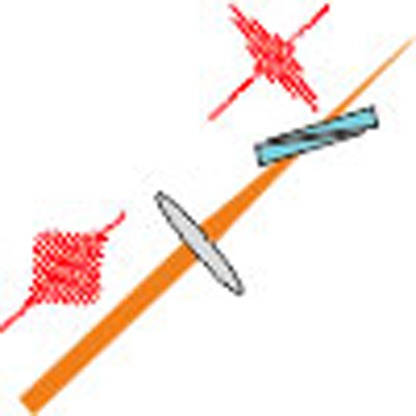

Ultrashort high-energy laser pulses in the 3–8 μm mid-infrared spectral range are of particular interest because of their role in high harmonic generation^1^,^2^,^3^, filamentation^4^,^5^,^6^, unusual regimes for nonlinear optical phenomena^7^ and particle acceleration^8^. These applications benefit from the extended oscillation period of the driving electromagnetic field and require, besides the long carrier wavelength, an ultrashort pulse duration of just a few optical cycles and a high pulse energy reaching into the multi-millijoule range. Attaining all three of these conditions simultaneously is one of the primary challenges of ultrafast laser technology. The limited gain bandwidth of optical amplifiers triggered the invention of various types of external pulse compression approaches based on nonlinear-optical spectral broadening and either subsequent or simultaneous dispersion compensation, referred to, respectively, as post-compression and nonlinear self-compression techniques. A powerful post-compression method for the generation of few-cycle pulses in the near IR at the sub-millijoule energy level is based on spectral broadening in a gas-filled hollow waveguide^9^,^10^,^11^ occurring in the regime of near-zero or slightly positive dispersion. In the negative dispersion regime, it is possible to realize a soliton-like nonlinear self-compression, whereby the negative group delay dispersion of an anomalously dispersive nonlinear medium counteracts the positive group delay dispersion arising from self-phase modulation (SPM). The solitonic type pulse compression scenario is widely realized in waveguides^12^ and to an extent can also be applied to femtosecond filaments in gas, where a small central fraction of the beam can exhibit self-compression because of the plasma anomalous dispersion^13^,^14^,^15^,^16^, and to anomalously-dispersive bulk solids^17^,^18^. For solid media, the energy of the self-compressed pulses in the femtosecond range is limited to a few microjoules because the critical power of self-focusing, *P*_cr_, is at the MW level. For millijoule pulses, relevant to most high-field laser pulse applications, the peak power *P* exceeds *P*_cr_ by several orders of magnitude. Propagation of a beam carrying pulses with high *P*/*P*_cr_ ratios over an extended distance in a nonlinear medium results in a rapid beam disintegration into multiple filaments and an overall loss of coherence that makes such pulse sources unusable for applications. The current literature therefore clearly indicates that upscaling filament-like regimes in condensed media for pulse self-compression beyond energies of several microjoules is not a viable route for generating few-cycle coherent high-energy pulses.

Here we report the experimental realization of self-compression in anomalously dispersive transparent solids of femtosecond mid-infrared pulses with energies reaching above 20 mJ, which represents a robust and reliable way to produce few-cycle optical pulses with peak powers exceeding 440 GW. The solitonic scenario is verified by extensive three-dimensional (3D) numerical modelling which reveals that under our operating conditions, the length of temporal compression is much shorter than the length of spatial modulation instability and beam breakup into multiple filaments. It is this separation of length scales, attainable in the regime of strong anomalous dispersion for our mid-infrared pulses, which allows us to use a solid medium for temporal compression at remarkably high energies. Whereas the merits of bulk anomalous dispersion for soliton-like self-compression in the mid-infrared have been already duly recognized and exploited in the microjoule pulse energy range^15^,^16^,^18^, in this work we demonstrate that the fundamental limitation imposed by the critical power of self-focusing can be surpassed by multiple orders of magnitude.

## Results

### Characterization of self-compressed pulses

**Figure 1:**
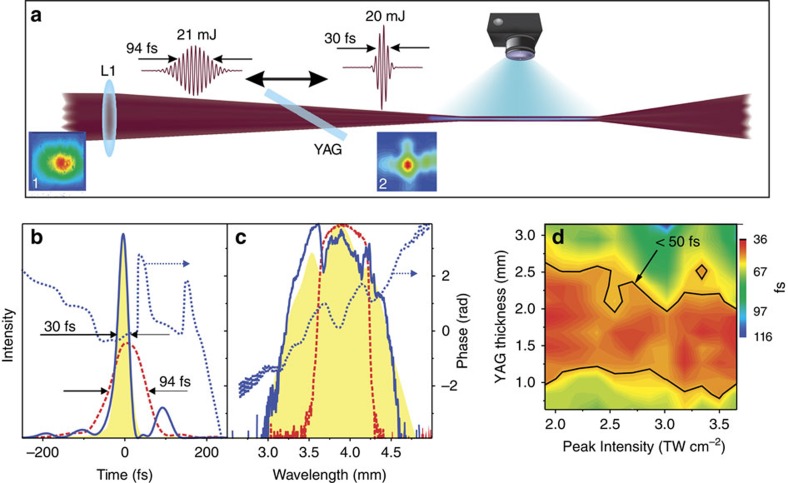
Setup for self-compression and characterization of mid-infrared pulses. (**a**) Sketch of the experimental setup: three-fold self-compression of 94 fs, 21 mJ pulses, centred at 3.9 μm, is achieved in a 2-mm thick YAG plate placed at a certain distance from the focusing lens L1; luminescence from filaments generated by the self-compressed pulses in air at ambient pressure is recorded by a digital camera; in the bottom beam profiles on the focusing lens (1) and on the output surface of YAG plate (2) are shown; (**b**,**c**) retrieved from SHG FROG measurements temporal (**b**) and spectral (**c**) pulse profiles of the output of 3.9-μm OPCPA system (dashed red line) and self-compressed in YAG pulses (blue solid line); note that in order to highlight the broadening of the spectrum ordinate axis in (**c**) is plotted on a logarithmic scale; area under the temporal pulse profiles shown in (**b**) correspond to the pulse energy. The yellow area represents calculated temporal profile and spectrum of the self-compressed pulse (normalized intensity). Dotted blue lines show retrieved temporal and spectral phases; (**d**) 3D-map representing the dependence of the output pulse duration on the thickness of the material and on the input peak intensity.

Second harmonic generation frequency resolved optical gating (SHG FROG) characterization of 21-mJ 3.9-μm pulses before and after self-compression are shown in Fig. 1. The self-compression takes place in a 2-mm thick yttrium aluminium garnet (Y_3_Al_5_O_12_, YAG) plate oriented at the Brewster angle and placed at a distance of 50 cm from the focal plane of a lens with the focal distance of 75 cm. For the characterization a fraction of the radiation is picked up by a CaF_2_ wedge directly after the YAG plate and directed into the SHG FROG apparatus and spectrometer. Further details of the experimental arrangement are given in Supplementary Methods and Supplementary Figs 1–4. As can be seen from Fig. 1b spectrally broadened due to SPM in YAG 94-fs pulses are self-compressed by a factor of 3 to 30 fs, which at 3.9 μm wavelength corresponds to less than 3 optical cycles. Note that in order to highlight the broadening of the spectrum ordinate axis in Fig. 1c the spectra are plotted on a logarithmic scale. We underscore that the SHG FROG measurement was performed on the whole beam, that is, without selecting a certain fraction along the transversal coordinate. The transform-limited pulse width supported by the entire spectrum is 26 fs (Supplementary Fig. 4d), which is only 11% shorter than the experimentally determined pulse width, indicating that the residual phase is quite small. As seen from the measured dependence of the pulse duration on the input intensity and on the YAG thickness (Fig. 1d), self-compression can be achieved in rather broad parameter range, which indicates insensitivity of the degree of self-compression to small-scale laser fluctuations. The energy of self-compressed pulses exceeds 19.7 mJ, indicating a >93% combined transmission efficiency.

Because of the Brewster angle incidence the ellipticity of the beam (for details see Supplementary Methods section) on the input surface of YAG plate was enlarged with beam diameters being 0.6 and 0.2 cm which resulted in the input intensity of <2.5 TW cm^−2^. It is important to notice that the intensity of 2.5 TW cm^−2^ in the case of 100 fs pulses corresponds to an energy density of <0.25 J cm^−2^ which is far below the optical damage threshold of both bulk YAG and common dielectric antireflection (AR) coatings which allows to use AR-coated YAG plates at normal incidence geometry for the reflection-lossless self-compression.

### Uniformity of the compression

We have also studied the uniformity of the compression across the beam profile. It follows from our numerical calculations the central part of the beam self-compresses more as compared to the overall beam but with an overall variation of the pulse duration that is less than ∼30% and across radial coordinate remains very smooth and uniform (as opposed e.g., to the strong pulse splitting observed in filamentation). This is supported by the experiments during which SHG FROG traces were recorded while selecting a fraction of the beam with an aperture which was placed right after the wedge *W3* (Supplementary Fig. 1). The results of the measurements presented in the Supplementary Fig. 5 reveal that, as predicted by the theory, pulse duration across the beam varies from 30 to 38 fs which corresponds to the central part of the beam and to the part corresponding to a 1/e intensity level correspondingly. Furthermore, as it can be seen from the right side of the Supplementary Fig. 5 the spectral broadening also varies across the beam accordingly with the spectrum featuring some modulations which are characteristic for SPM in a dispersive medium.

### Determination of the origin of losses

The origin of the 7% losses was clarified by measuring the dependencies of the transmission on the incident intensity at different material thicknesses (for details see Supplementary Methods and Supplementary Fig. 6 in particular). As expected, at low incident intensity <0.1 TW cm^−2^, which was achieved by detuning the compressor, the transmission is 100%. In the case when the compressor is optimized and the input intensity is in the range 0.5 TW cm^−2^<I<1.5 TW cm^−2^ the transmission stays at the level of 97% and is independent on the material thickness in the range of 1–3 mm. This reveals that the 3% losses are due to plasma formation in air in the focus of the 75-cm lens. With further increase of the intensity, the transmission decreases with the steeper decrease at larger material thickness, indicating ionization and/or induced absorption losses. This implies only <4% losses in the YAG crystal and reveals that the energy of self-compressed pulses exceeds 20 mJ.

### Focusability of self-compressed pulses

Focusability of self-compressed pulses was studied by a knife-edge method (see Supplementary Methods and Supplementary Fig. 7). In the near field the beam profile in the case of self-compression is transformed to a Bessel-like (Supplementary Fig. 7c) (from a close to Gaussian shape in the absence of self-compression (Supplementary Fig. 7d)). Without any additional optical elements added, the transformation causes a nearly two times increase of the beam waist size on the one hand, and 3-fold enlargement of the Rayleigh length, on the other (Supplementary Fig. 7a). Since the beam waist can be externally controlled by a harder/softer focusing, it is difficult to evaluate an impact of the self-compression on achievable intensity. An increased Rayleigh range resulting into longer interaction range, in turn, might be beneficial for some applications.

### Numerical modelling

**Figure 2:**
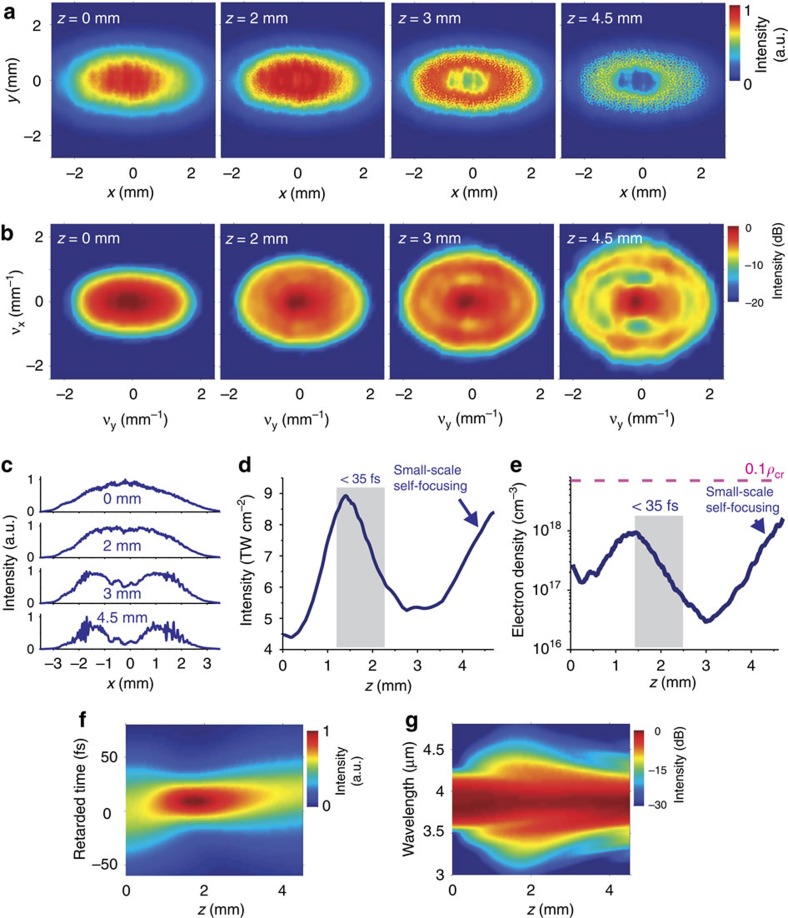
Three-dimensional simulations for the dynamics of the mid-infrared beam in YAG. (**a**) evolution of the beam profile with propagation in YAG plate (*z* direction); (**b**) evolution of the angular spectrum (Fourier transform of the beam profile) with propagation in YAG plate, *ν*_x_ and *ν*_y_ are the spatial frequencies; (**c**) 1D cuts of beam dynamics; (**d**) the field intensity *I*_m_, found as the maximum intensity over the beam; (**e**) the maximum electron density across the beam calculated as functions of the propagation coordinate *z*; ρ_cr_ stands for the critical plasma density; (**f**) evolution of the temporal pulse profile during propagation in YAG; (**g**) evolution of the spectrum during propagation in YAG.

For a quantitative description of the separation of different nonlinear length-scales, we compute the transverse field intensity profiles for different propagation lengths *z* (Fig. 2). For the chosen set of parameters, the shortest pulse width of self-compressing soliton transients is achieved at *z*≈1.45 mm. At this point of maximum pulse compression, the spectral broadening is largest (Fig. 2g) and the intensity, as can be seen from Fig. 2d, reaches its local maximum. Analysis of the beam profile and angular spectrum of the field at this point shows that, although the modulation instabilities start to build up, the beam does not display any noticeable degradation of its intensity profile (Fig. 2a–c). Beyond *z*>3 mm, however, despite pulse broadening (Fig. 2f) and spectral narrowing and split-up (Fig. 2g), the intensity rapidly raises and is accompanied by the buildup of hot spots across the beam as a result of multiple filamentation, leading to a dramatic degradation of the beam profile and angular spectra (Fig. 2a–c). Consequently, the zone of maximum pulse compression (Fig. 2d) is distinctly separated (in propagation distance) from the region where the beam starts to break up into multiple filaments (that is, small-scale self-focusing^19^ takes place), thus allowing pulse self-compression to be implemented without the detrimental beam break up that should otherwise be expected at such high powers.

## Discussion

The full 3D numerical model (see Methods section) was first used to reproduce the pulse transformation observed in the experiments. Figure 1b,c shows the simulation results (contours shaded in yellow) superposed on the experimental results (blue lines). The good quantitative agreement indicates the accuracy of the numerical simulations. The key tendencies of these transformations appear to be very similar to the generic soliton pulse self-compression scenario. However, we underline that the overall picture of pulse compression in a focused high-power beam as implemented in our experiment is much more intricate and cannot be reduced to a one-dimensional textbook soliton dynamics^20^. Most importantly, in a full 3D propagation the spatial dynamics gains critical importance. In typically studied scenarios, spatiotemporal filamentation dominates the pulse dynamics with simultaneous temporal compression and spatial collapse followed either by temporal pulse splitting or by multiple refocusing cycles. Furthermore, at *P* merely several times over the threshold power of self-focusing, *P*_cr_, the spatial modulation instability and consequent spatial breakup into multiple filaments becomes imminent with propagation^19^. Here we have showed, both experimentally and numerically, that in the case of mid-infrared pulses even at peak powers four orders of magnitude above *P*_cr_ it is nevertheless possible to suppress the above-mentioned types of unwanted dynamics.

Our numerical simulations also predict that similar situation holds for another dielectric transparent in the mid-infrared, namely CaF_2_. We experimentally demonstrate (Supplementary Fig. 8) that similar-level self-compression can be achieved in a pair of CaF_2_ lenses resulting in slightly divergent beam, that is, without any beam waist as in the case of compression in converging beam in the YAG plate. Next to obvious practicality (self-compression can be achieved without use of any specially designed optical elements rather than conventional lenses) this reveals two important points: first, a moderate wave-front curvature (positive or negative) does not play any crucial role in self-compression, and, second, the use of negative (concave) lens as self-compression medium is profitable because of opposite signs of beam intensity and material-thickness gradients.

The ionization losses in our experiments (4%) was found to be lower than an ionization losses of 15% observed in the earlier experiments^18^ on mid-infrared superconcontinuum generation in solids. While in that paper^18^, the beam undergoes a full focusing–defocusing cycle in a solid-state plate, in our experiments a much broader driver beam only acquires a small wave-front curvature as it propagates through the YAG plate. As a result, the electron density in the solid-state medium in our experiments is noticeably lower than the electron density under conditions of reference^18^. As can be seen in Fig. 2e, a typical electron density on the axis of the mid-infrared driver in our work is 10^17^−10^18^ cm^−3^, which is almost an order of magnitude lower than the electron density reported by Hemmer *et al*.^18^.

**Figure 3:**
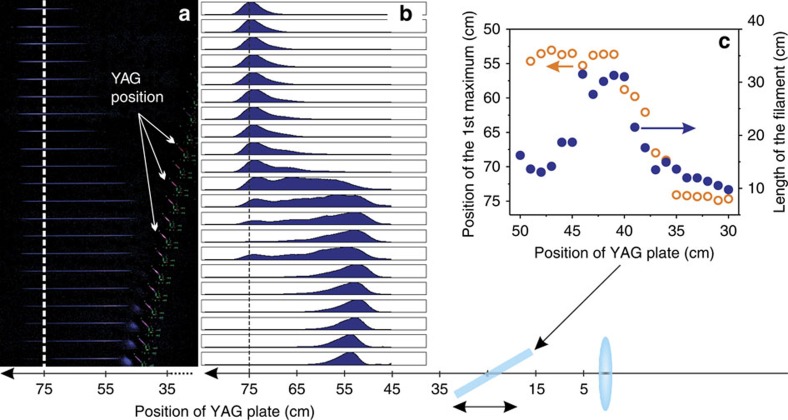
Filamentation of self-compressed pulses in ambient air. (**a**) Photos of the visible part of the filament in air at different distance between the 75-cm lens and Brewster-oriented 2-mm thick YAG plate; direction of light propagation is indicated by an arrow; YAG plate (position of the plate is indicated by the luminescence) was moved from the lens towards the focus which corresponds to the photo order from top to bottom; dashed line indicates the focal plane of the 75-cm lens. (**b**) Longitudinal intensity distribution in a filament as extracted after digital processing of the photos shown in (**a**); dashed line indicates the focal plane of the 75-cm lens. (**c**) Dependences of the length of the filament (at 1/e^2^ level) and of the position of the first (closest to the focusing lens) maximum of the luminescence originating from a filament on the distance between the 75-cm lens and Brewster-oriented 2-mm thick YAG plate.

As a proof of concept in the strong-field regime, directly sensitive to the scaling of the peak power boosted by the nonlinear pulse self-compression, we examined filament formation in air behind a 2-mm thick Brewster-oriented YAG plate placed at different distances from the *f*=75 cm lens (Fig. 3). To monitor the influence of pulse self-compression on filament formation the energy of pulses was decreased to the level of 10 mJ. At pulse energies above 20 mJ complicated filament shapes were detected even in the absence of self-compression. The observed filamentation in ambient air is a convincing evidence for a dramatically increased peak power of the self-compressed pulse as well as a proof of high pulse and beam fidelity. Furthermore, the position of the first maximum of luminescence moves towards the focusing lens with increasing of pulse peak power (Fig. 3c), which is in agreement with the generally expected dynamics of self-focusing^21^,^22^. Detailed studies on filamentation of self-compressed mid-infrared pulses will be presented in a separate publication. Noteworthy, the simple in-line geometry of the demonstrated low-loss self-compression can be straightforwardly employed for strong-field applications.

The final point we would like to address in the discussion is related to pulse dynamics in the filament in air after the YAG plate. The peak power of the compressed pulse behind the YAG plate (>0.44 TW) exceeds substantially the critical power of self-focusing in air. As far as the dynamics of the mid-infrared pulse along the entire filament length in the atmosphere is concerned, it involves a dramatic spectral broadening (Supplementary Fig. 9). For the conditions of our experiments, however, this broadening does not translate into an equally dramatic pulse compression. Unfortunately, because of a multi-octave spanning spectrum experimental characterization of temporal pulse modification in the filament is rather complicated. Our simulations (Supplementary Fig. 9), however, reveal a pulse width of ∼28 fs behind the filament, that is, the mid-infrared waveform is shortened by additional 7% from the 30-fs duration measured at the output of the YAG plate.

In conclusion, reports on self-compression in solid crystals were previously limited to pulse energies of several few micro-Joules due to physical limitations leading to catastrophic pulse breakup at higher energies. For the mid-infrared pulse propagation in transparent dielectrics we show that it is possible to decouple the temporal and the spatial dynamics because the two exhibit distinctly different length scales. We then use this regime of propagation to achieve the 3-orders of magnitude increase in the compressed pulse energy, thus providing a platform for future strong-field studies in the mid-infrared region. We also demonstrate that this simple and robust technique of self-compression is applicable in a rather broad wavelength range (Supplementary Methods and Supplementary Figs 10 and 11) and potentially allows achieving multi-TW peak powers (Supplementary Methods and Supplementary Fig. 12).

## Methods

### Self-compression experiments

Sub-100 fs, 21-mJ pulses centred at 3.9 μm at a repetition rate of 20 Hz were generated by a hybrid OPA/OPCPA system^23^ based on Type II potassium titanyl arsenate (KTA) nonlinear optical crystals. Experimental setup designed for the investigation of self-compression of 3.9-μm pulses is presented in Supplementary Fig. 1.

### Filamentation

Photos of filaments ignited by 10-mJ, 3.9-μm pulses, manifested by luminescence of neutral and ionized molecular nitrogen, (Fig. 3a) were taken with stationary fixed digital photo camera (Canon 350D) with the integration time of 10 s as it is shown in Fig. 1a. For each photo YAG plate was moved away from the focusing lens with the step of 1 cm. Taken photos were processed by converting into gray-scale colour map, performing inverse gamma correction (in order to linearize intensity scale), cropping the area in the vicinity of the filament and integrating along the axis perpendicular to the direction of pulse propagation, which provides longitudinal intensity distribution in a filament (Fig. 3b). From the obtained data we have determined the dependence of the length of the filament (at the level of 1/e^2^), as well as of the position of the first (closest to the focusing lens) maximum of the luminescence on the distance between focusing lens and YAG plate.

### Modelling

To understand the spatiotemporal dynamics of ultrashort pulses behind the self-compression of multi-millijoule femtosecond mid-infrared pulses in transparent dielectrics, we performed numerical modelling using the three-dimensional time-dependent generalized nonlinear Schrödinger equation^24^,^25^ for the amplitude of the field, including all the key physical phenomena, such as dispersion and absorption of the medium, beam diffraction, Kerr nonlinearities, pulse self-steepening, spatial self-action phenomena, ionization-induced optical nonlinearities, as well as plasma losses and dispersion. The field evolution equation is solved jointly with the rate equation for the electron density, which includes impact ionization and photoionization with the photoionization rate calculated using the Popov–Perelomov–Terentyev version^26^ of the Keldysh formalism^27^. Simulations are performed for typical parameters of YAG crystal—a band gap of 6.4 eV, the Kerr-effect nonlinear refractive index *n*_2_=4 × 10^–16^ cm^2^ W^−1^, and the higher order Kerr effect (HOKE) coefficient *n*_4_=–3 × 10^–29^ cm^4^ W^−2^. The next-order terms (*n*_6_, *n*_8_…) are, however, negligibly small within the entire range of intensities studied in our experiments. Dispersion of YAG crystal was included in the model through a Sellmeier relation^28^. Spatial modulation instabilities leading to the formation of multiple filaments are seeded in our model by superimposing a Gaussian-noise modulation on the input beam profile^29^. Simulations were performed using an MPI parallel programming interface on the Chebyshev and Lomonosov supercomputer clusters of Moscow State University. We underline that the model includes all expected nonlinear effects including ionization and yet is able to precisely reproduce our experimental results only if we include a HOKE term. There has been significant discussion in the literature regarding the actual role and relevance of such terms—here the HOKE simply plays the role of an additional saturation term that appeared to be necessary in order to achieve full quantitative agreement with experiments and does not by itself represent a demonstration that HOKE is the mechanism through which this attained.

### Data availability

The data that support the findings of this study are available from the corresponding author on request.

## Additional information

**How to cite this article:** Shumakova, V. *et al*. Multi-millijoule few-cycle mid-infrared pulses through nonlinear self-compression in bulk. *Nat. Commun.* 7:12877 doi: 10.1038/ncomms12877 (2016).

## Supplementary Material

Supplementary Information Supplementary Figures 1-12, Supplementary Methods and Supplementary References.
