# Supplementary material for: Multi-millijoule few-cycle mid-infrared pulses through nonlinear self-compression in bulk
Source: Nat Commun. 2016 Sep 13;7:12877. doi: 10.1038/ncomms12877 (PMC5027276; doi:10.1038/ncomms12877)
Supplement: Supplementary Information — Supplementary Figures 1-12, Supplementary Methods and Supplementary References. [file ncomms12877-s1.pdf]

Supplementary Figures

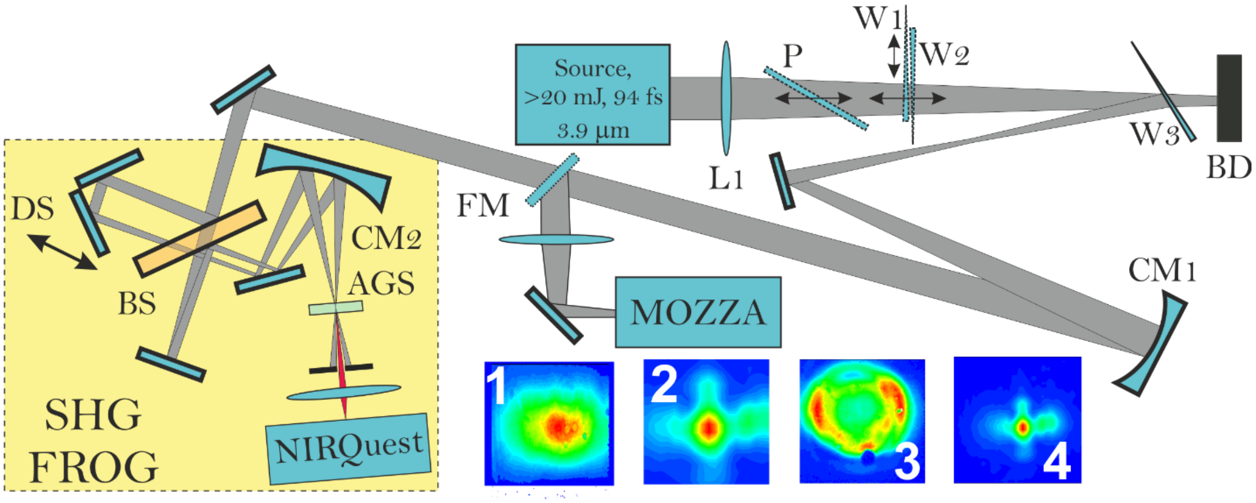

Supplementary Figure 1. Setup for the generation and characterization of self-compressed mid-IR pulses. L1 - CaF_2 lens with the focal length of 750 mm; P – 2-mm thick, Brewster angle oriented YAG plate; W1 and W2 - YAG wedges with the apex angle of 6 deg; W3 – CaF_2 wedge; BD – beam dump; FM – flip mirror; CM1 and CM2 - spherical mirrors with the radii of curvature 100 cm and 20 cm respectively; MOZZA – acousto-optic spectrometer operating in the spectral range 1-5 µm (FASTLITE); DS – motorized delay stage; BS – broadband pellicle beam-splitter; AGS – 0.2 mm-thick silver thiogallate nonlinear optical crystal; NIR-Quest – near-infrared spectrometer operating in the spectral range 0.9-2.5 µm (OceanOptics); The beam profiles presented at the bottom are taken with a CCD camera (Pyrocam): 1 – at the position of lens L1, 2 – by 2f-2f imaging of the output surface of W2, 3 – at the position of CM1, 4 – at the position of AGS crystal.

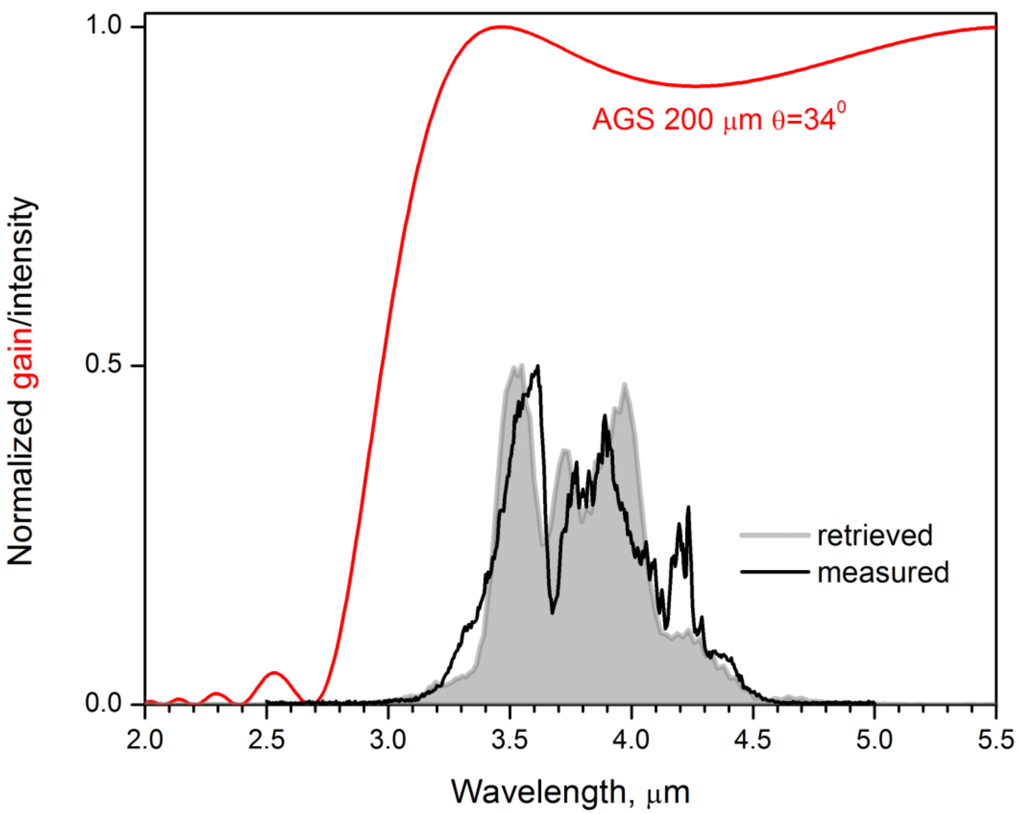

Supplementary Figure 2. Phase-matching bandwidth. Calculated phase-matching curve of 0.2-mm thick Type I AGS crystal in the case of theta angle of 34 deg and measured and retrieved spectra of the self-compressed pulses.

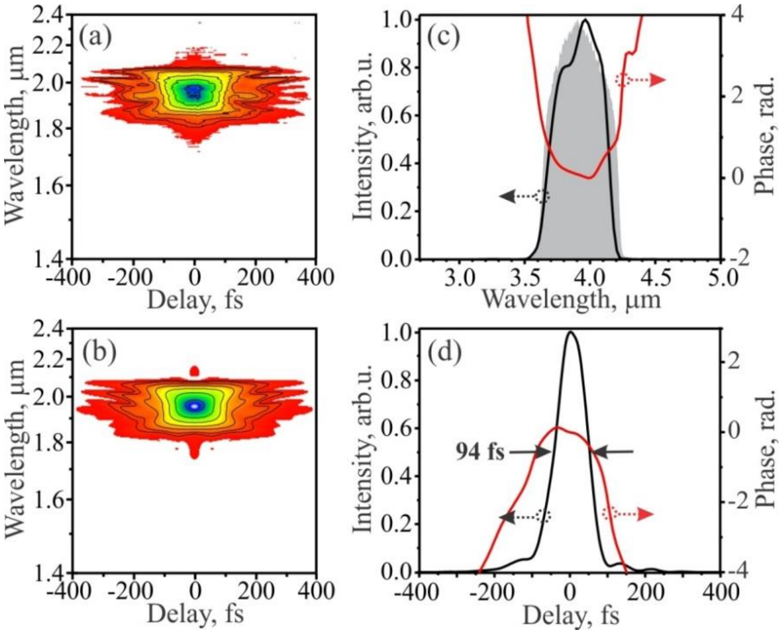

Supplementary Figure 3. Characterization before self-compression. SHG FROG characterization of the output of OPCPA before self-compression: measured (a) and retrieved (b) SHG FROG traces; (c) retrieved (black solid line) and measured with MOZZA (gray area) spectra; by red line retrieved spectral phase is drawn; (d) retrieved temporal pulse profile (black solid line); by red line retrieved temporal phase is drawn; grid size is 512; retrieval error is 0.0036.

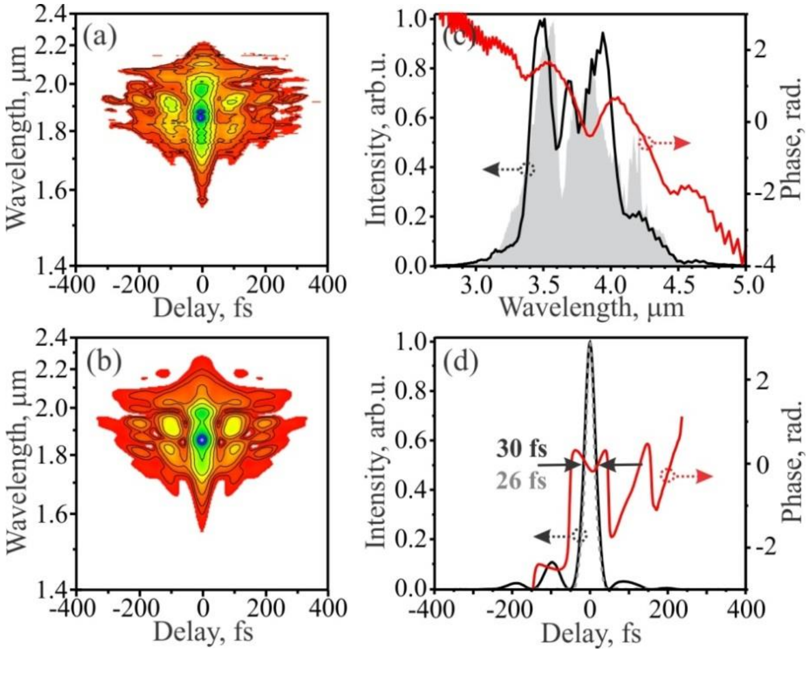

Supplementary Figure 4. Characterization after self-compression. SHG FROG characterization of self-compressed pulses: measured (a) and retrieved (b) SHG FROG traces; (c) retrieved (black solid line) and measured with MOZZA (gray area) spectra; by red line retrieved spectral phase is drawn; (d) retrieved temporal pulse profile (black solid line) and calculated transform limited pulse profile (dashed line); by red line retrieved temporal phase is drawn; grid size is 512; retrieval error is 0.0056.

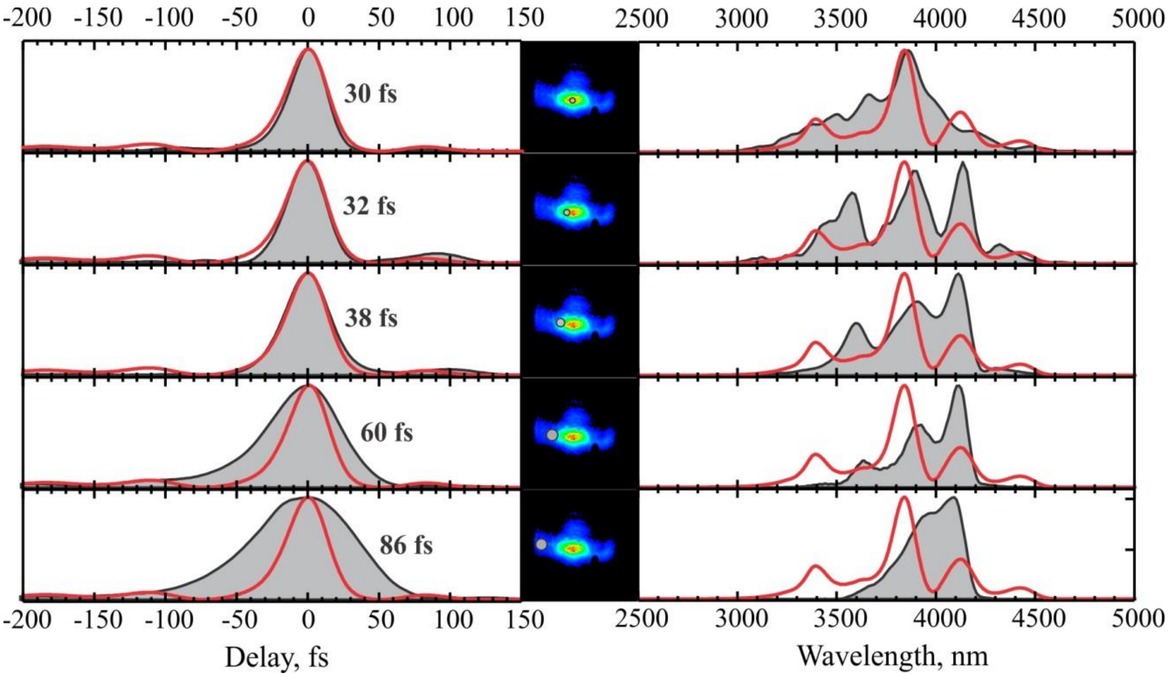

Supplementary Figure 5. Homogeneity of self-compression. Examination of self-compression across the beam by selecting a part of the beam by an aperture as indicated by a gray area in the middle panels. The area corresponds to the size of the aperture. Left panels: retrieved from SHG FROG measurements temporal pulse profiles. Right panels: retrieved from SHG FROG measurements spectra. By red lines in the left and right panels temporal profiles and spectra of the entire beam are drawn.

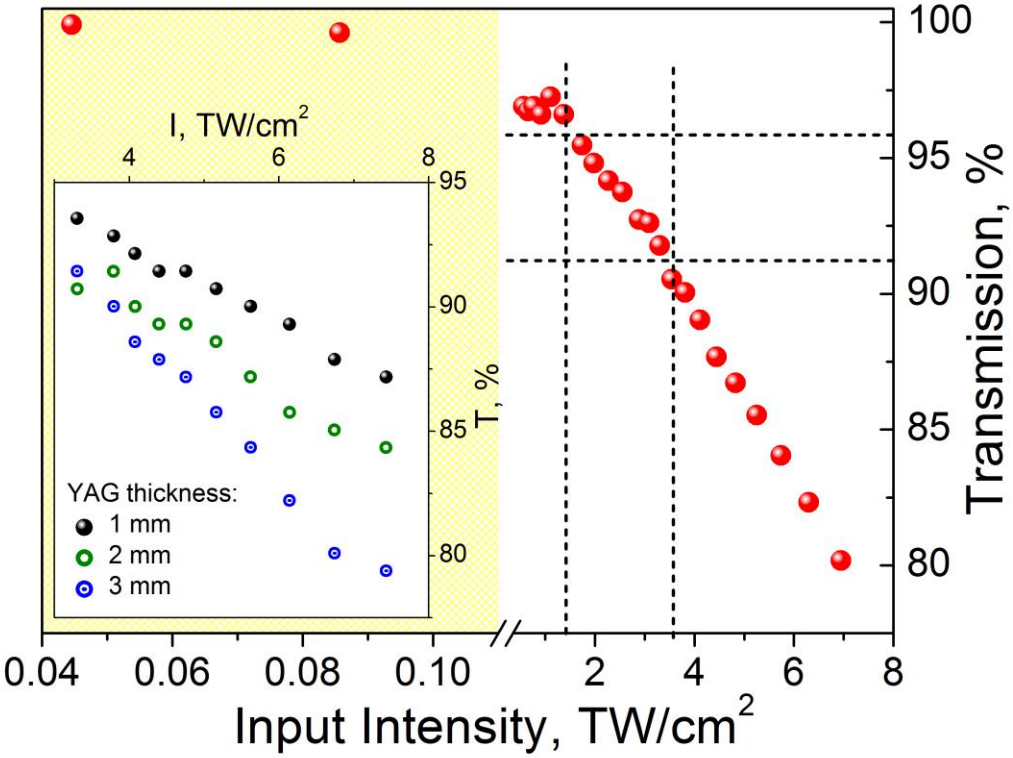

Supplementary Figure 6. Origin of losses. Red solid dots: dependence of the optical transmission on the input intensity measured for a 2-mm thick YAG plate at normal incidence (after accounting for Fresnel reflection losses; for the details see text); dashed lines bounder the region of efficient self-compression in 2 mm of YAG; the area shaded in yellow displays transmission measurements where the input intensity was controlled by detuning the pulse compressor of the OPCPA from the highest compression ratio; the inset shows the intensity dependent transmission of a pair of YAG wedges for three different thicknesses of material indicated in the figure.

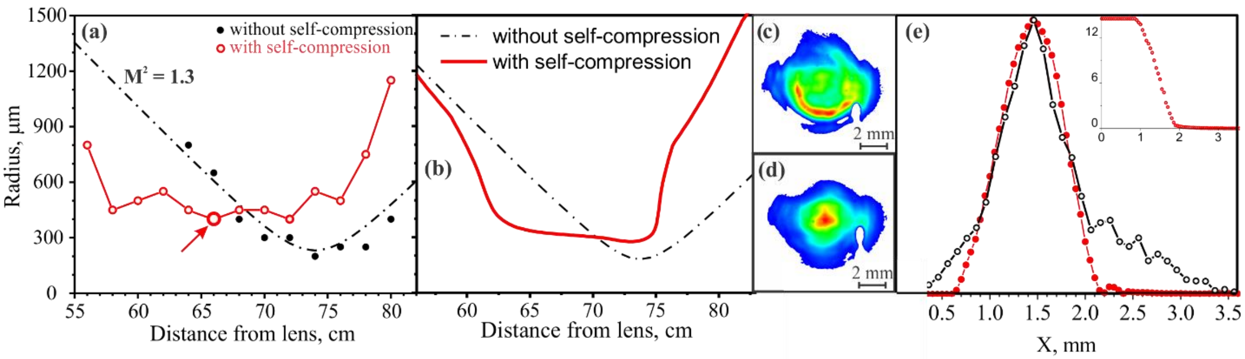

Supplementary Figure 7. Transformation of beam profile and focusability. (a) Examination of the beam waist after the self-compression in 2-mm thick YAG plate (red open circles) and in the absence of YAG plate in the beam (black solid); the knife-edge experiments were performed in a beam attenuate by taking a double reflection from CaF_2 wedges; dash-dotted black line is a fit revealing M^2 value of 1.3 in the case of absence of YAG plate; (b) simulated beam waists at the focus of a 75-cm lens with (solid line) and without (dash-dotted line) a 2-mm-thick YAG plate placed at a distance of 45 cm from the lens; (c) and (d) beam profiles measured with a pyroelectric camera (Pirocam II) at a distance of 45 cm from the focus (120 cm from the lens) in the presence (c) and absence (d) of YAG plate; red arrow in (a) indicates the position at which transverse intensity distributions shown in panel (e) were determined by a knife-edge method (red solid dots) and pyroelectric camera (open black dots.)

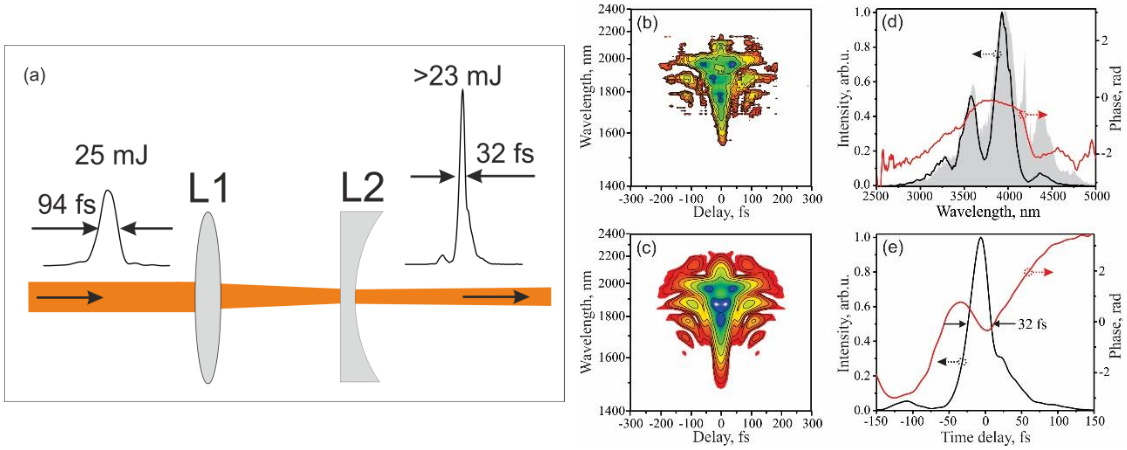

Supplementary Figure 8. Self-compression in a pair of CaF_2 lenses. (a) Realization of self-compression in a pair of CaF_2 lenses. Pulse profiles are as extracted from SHG FROG measurements. The areas under the pulse envelopes are scaled proportionally to the pulse energy; (b) measured SHG FROG trace; (c) retrieved SHG FROG trace; (d) measured (gray filled area) and retrieved (solid black line) spectra and spectral phase (red line); (e) retrieved temporal pulse profile (black line) and phase (red line).

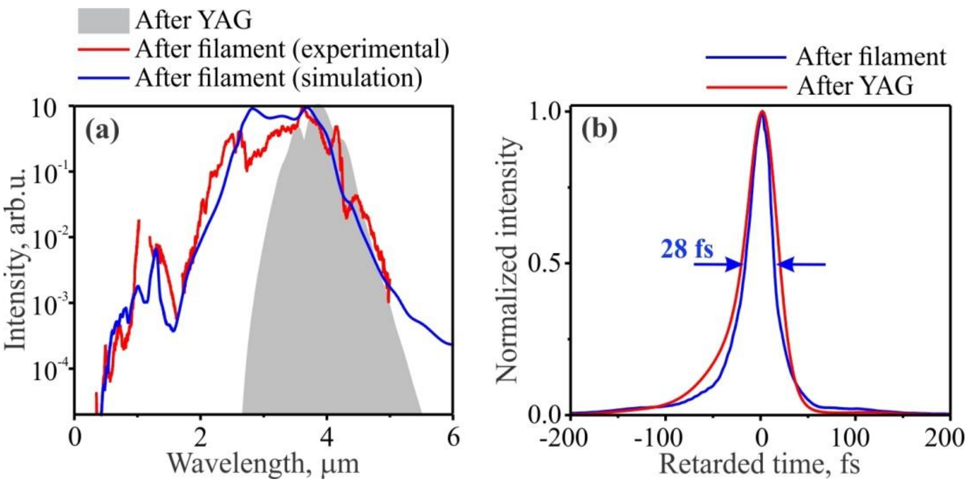

Supplementary Figure 9. Transformations during filamentation in air. (a) Measured spectrum after YAG plate and measured and simulated spectra after filamentation in air in the case of self-compression in YAG; (b) simulated temporal pulse profiles after YAG plate and after filamentation in air.

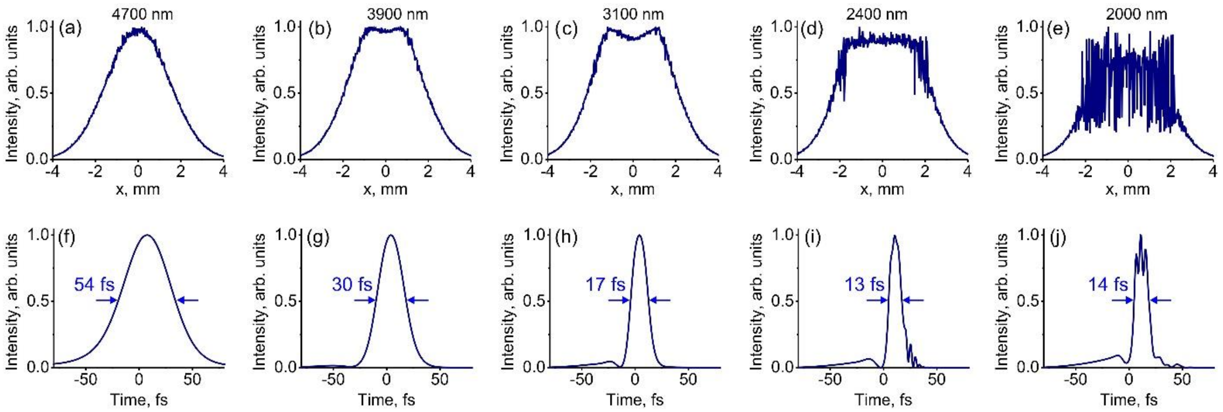

Supplementary Figure 10. Wavelength scalability of self-compression (1). Calculated transverse beam profiles (a - e) and temporal pulse shapes (f - j) at the output of YAG plate with a thickness, corresponding to maximum self-compresion, of 1.1 mm (a, f), 2 mm (b, g), 2.7 mm (c, h), 3.3 mm (d, i), 3.6 mm (e, j). The field intensity at the input surface of the plate of 4.0 TW/cm^2 was kept constant for all the wavelength. The YAG plate is located at a distance of 50 cm from a 75-cm-focal-length lens. Simulations were performed for the central wavelength of 4.7 μm (a, f), 3.9 μm (b, g), 3.1 μm (c, h), 2.4 μm (d, i), and 2.0 μm (e, j).

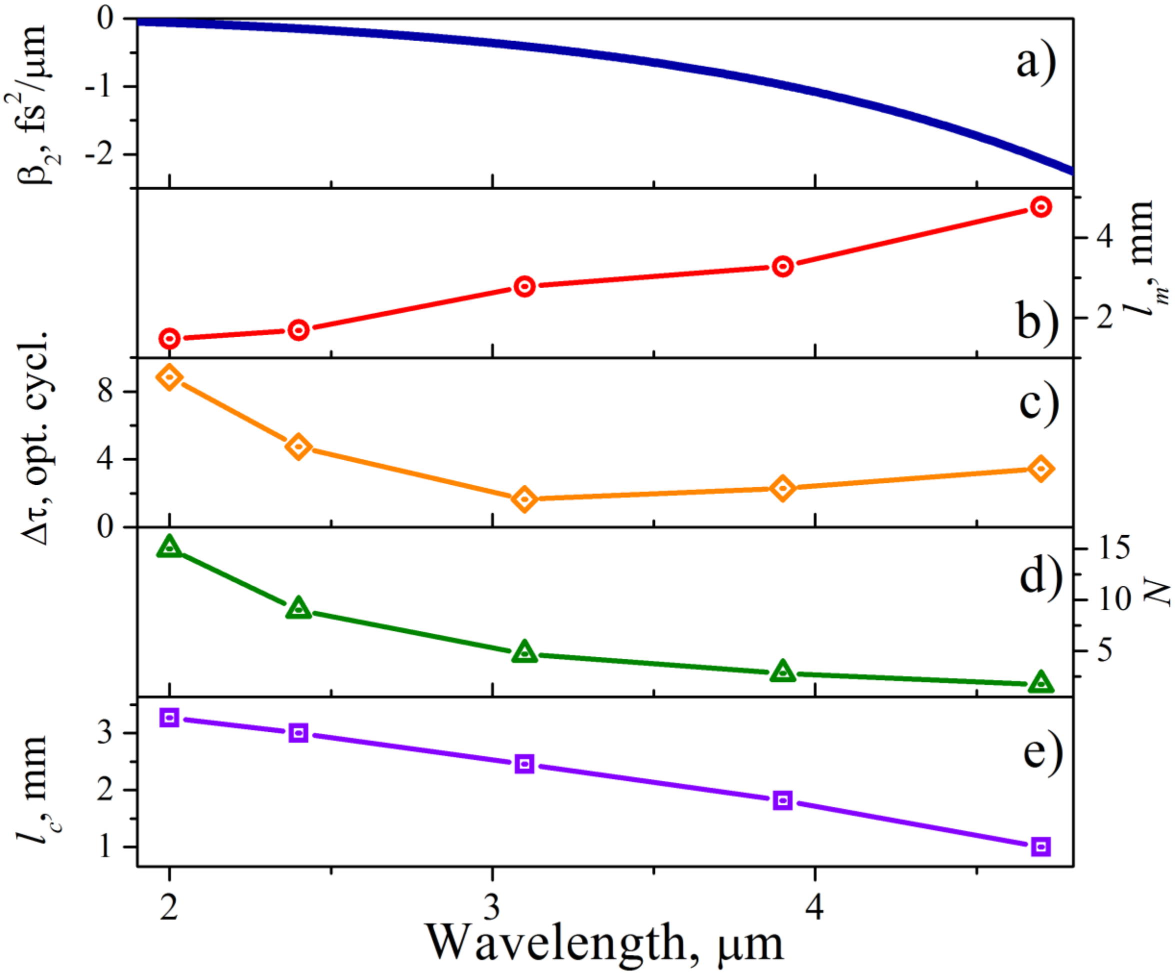

Supplementary Figure 11. Wavelength dependence of self-compression parameters. Calculated for YAG wavelength dependences of the group-velocity dispersion (a), modulation instability length l_m (b), pulse width at a propagation distance $z = \min\{l_c, l_m\}$ (c), soliton number N (d) and self-compression length l_c (e) for a laser pulse with an input pulse width of 80 fs and the initial field intensity of 4 TW/cm^2.

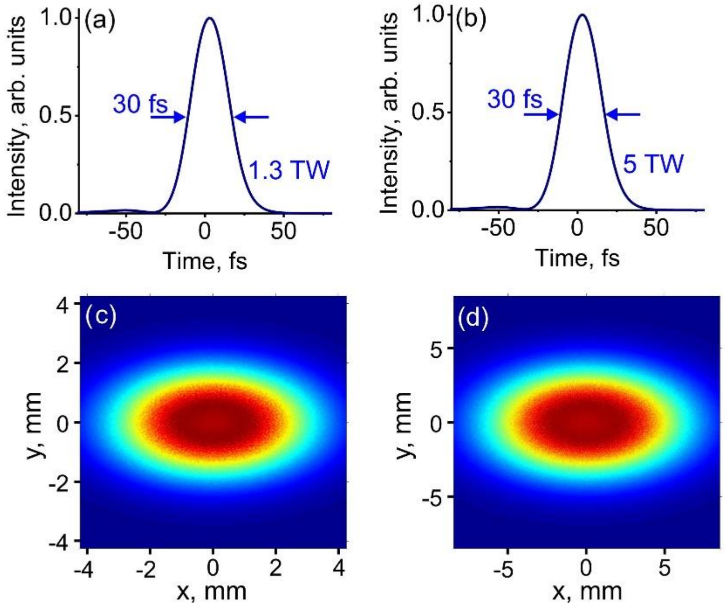

Supplementary Figure 12. Energy scalability of self-compression. Calculated temporal pulse shapes (a, b)s and spatial beam profiles (c, d) at the output of a 2-mm-thick YAG plate. The beam is focused to yield field intensity of 4.5 TW/cm^2 at the entrance surface of the plate. The input energy is 43 mJ (a, c) and 160 mJ (b, d). The input pulse width is 94 fs, the central wavelength is 3.9 μm. The peak power of the compressed pulse is 1.3 TW (a, c)and 5 TW (b, d)

Supplementary Methods. Sub-100 fs pulses centered at 3.9 µm (second-harmonic-generation frequency-resolved optical gating (SHG FROG) characterization is given in the Supplementary Fig.3) at a repetition rate of 20 Hz were generated by a hybrid OPA/OPCPA system based on Type II potassium titanyl arsenate (KTA) nonlinear optical crystals. The three-stage OPA and three-stage OPCPA are respectively pumped by 1-mJ, 200-fs Yb:CaF_2 and 1-J, 100-ps Nd:YAG laser systems. Both femtosecond and picosecond laser amplifiers are seeded by a common Yb:KGW laser oscillator, which ensures all-optical synchronization of the OPA and OPCPA units. The OPCPA is seeded by stretched in a GRISM stretcher signal pulses centered at 1460 nm, originating from femtosecond OPA; the idler 3.9-µm pulses generated in the second and amplified in the third OPCPA stage are compressed to sub-100 fs pulse duration in a diffraction-grating based compressor. The energy of compressed pulses exceeds 20 mJ.

Experimental setup designed for the investigation of self-compression of 3.9-µm pulses is presented in the Supplementary Fig.1. Compressed in a grating compressor sub-100 fs pulses were focused by a plano-convex CaF_2 lens L1 with the focal length of 75 cm. Here we would like to stress that the particular focal length of the lens of 75 cm is nonessential for self-compression, this focal length was chosen for convenience of easy tuning of the intensity. A pair of YAG wedges (W1, W2) with the apex angle of 6° or, alternatively, a 2-mm-thick YAG plate oriented at Brewster's angle were inserted into the beam. The collimated beam after the OPCPA (at the output of the compressor) is slightly elliptical with radii at FWHM level being $r_x = 4.5\ mm$, $r_y = 2.9\ mm$. Astigmatism introduced by a 2 mm thick Brewster plate, expressed as longitudinal distance between the sagittal and tangential foci, in the case of 75-cm focal length lens is 0.8 mm which corresponds to an added astigmatic lens with the focal distance of -91m, which is a negligible value. We also estimate a spatial chirp introduced by the Brewster angle oriented YAG plate: spectral components corresponding to 3 µm and 5 µm wavelength after passing through 2-mm thick Brewster angle YAG plate are separated by about 26 µm, which might be not negligible value in the case of tight focusing. However, since the energy density on the YAG plate is $<0.25\ J/cm^2$, an AR coated YAG plate at normal incidence can be used for achieving the self-compression without reflection losses and without introduction of spatial chirp.

The intensity in YAG was varied by changing the distance between the wedge-pair (or the YAG plate) and the lens L1. The curvature of the wave front induced by the long-focal-length lens used in experiments has virtually no influence on the self-compression scenario. With the curvature of the wave front included in simulations, the field intensity right behind the YAG plate is only 1.6% higher than the field intensity at the same point in calculations performed for a plane wave front. This slightly higher field intensity in simulations for a converging beam translates into a 0.3% shorter pulse and a 0.2% shorter pulse compression length. The thickness of YAG material was controlled in a continuous way by varying the insertion depth of the wedges.

Self-compressed pulses were characterized by the SHG FROG based on a Type I 0.2 mm-thick silver thiogallate (AGS) nonlinear optical crystal, which assures phase matching bandwidth from 3 µm to 5 µm (Supplementary Figure 2) and a near-infrared spectrometer (NIRQuest, OceanOptics). Two pulse replicas in the SHG FROG apparatus were produced by a broadband

2-µm-thick pellicle beam splitter (Thorlabs). To equalize the spectra of both replicas, the beams were once transmitted through and once reflected by the beam splitter. Pulses directed to the SHG FROG were attenuated by reflecting approximately 1.5% of the total energy by a CaF_2 wedge W3. The wedge W3 was placed at a distance of a few centimeters from the YAG plate, consequently, any significant effects of nonlinearity in air can be neglected due to small interaction length (distance between YAG plate and the wedge W3 was L=3 cm) and relatively large beam size. We estimate that in the case when the intensity of self-compressed pulses at the output of YAG plate is $I=10\ TW/cm^2$, B-Integral $B=2\pi/\lambda\ n_2\ L\ I$ does not exceed value of 0.1. Here $n_2=1.9x10^{-7}\ cm^2/TW$ is the nonlinear refractive index of air[1]. Pulse self-compression presented here is thus totally due to the nonlinear pulse dynamics in the YAG plate.

Spatial-temporal distortions of the self-compressed pulses were circumvented by a 4-f imaging of the output surface of the wedge W2 (YAG plate) on the surface of AGS crystal which was performed by a pair of spherical mirrors CM1 and CM2 with the radii of curvature of 100 cm and 20 cm. The 4-f imaging resulted in 5× reduced size of the spot on the AGS crystal as compared to the size on the W2 (YAG plate). The spectra of mid-IR pulses were recorded by acousto-optic based scanning spectrometer (MOZZA, FASTLITE)[2].

Homogeneity of self-compression across the beam was examined by cutting a fraction of the beam by an aperture placed right after the wedge W3. The diameter of the aperture was changed from 1 mm in the center of the beam to 1.8 at the edge (as it is shown in Supplementary Fig.5).

Focusability of the self-compressed pulses was examined by a knife-edge method: a sharp metal blade was moved across the beam by rotating a micrometer screw; the energy of the transmitted light was recorded with a liquid nitrogen cooled InSb detector and a digital oscilloscope. In order to avoid a damage of the knife the measurements were performed in a weak beam: the light reflected by a wedge W3 (Supplementary Fig.1) was additionally attenuated by taking one more reflection from CaF_2 wedge.

Determination of the stability of spectral broadening during self-compression. Stability of the spectra after the self-compression in bulk crystal was investigated by recording second harmonic spectra with NIRQuest spectrometer (OceanOptics) operating in a single shot mode. Second harmonics was generated because of the absence of mid-IR spectrometer capable of operating in a single shot mode. Since the stability of the second harmonics generation in the case of femtosecond pulses is influenced by both spectral intensity and spectral phase fluctuations, we believe that measured error values represent a top margin of the spectral instability.

For the evaluation of spectral stability we recorded 1000 spectra of seconds harmonics generated in a 0.2-mm thick AGS crystal. Standard deviation was calculated using the formula:

$$\sigma = \sqrt{\frac{1}{(N-1)}\sum_{i=1}^{N}(S_i - S_{av})^2}$$

where N – is the number of measurements (N = 1000), S_i is the spectrum recorded at the ith measurement, S_{av} is the averaged spectrum. A standard error is related to the standard deviation by the following relation:

$$\mathrm{SE} = \frac{\sigma}{\sqrt{\mathrm{N}}}$$

Obtained results reveal that at the intensity level $I>1/e^2$ the standard error is $SE<3\%$ while at the wings of the spectrum ($I<<1/e^2$) the error is substantially higher which partially is because of the dark noise of the spectrometer.

Determination of the origin of losses. The origin of the 7% losses was clarified by measuring the dependencies of the transmission on the incident intensity at different material thicknesses (Supplementary Fig.6). In order to have an adjustable material thickness we used a pair of YAG wedges (W1 and W2), shown in Supplementary Fig.1, the YAG wedges were placed at close to normal incidence with respect to the laser beam to have maximum accessible range of the material thickness. Supplementary Fig.6 shows the transmission dependence corrected for the Fresnel losses on the YAG surfaces ($n=1.757$). As expected, at low incident intensity below 0.1 TW/cm^2, which was achieved by detuning the compressor, the transmission is 100%. In the case when the compressor is optimized and input intensity is in the range $0.5\ TW/cm^2<I<1.5\ TW/cm^2$ the transmission stays at the level of 97% and is independent on the material thickness in the range of 1-3 mm. This reveals that the 3% losses are due to plasma formation in air in the focus of the 75-cm lens. With further increase of the intensity, the transmission decreases with the steeper decrease at larger material thickness (inset in Supplementary Fig.6), indicating ionization and/or induced absorption losses. This implies only <4% losses in the YAG crystal and reveals that the energy of self-compressed pulses exceeds 20 mJ.

Self-compression in a pair of CaF_2 lenses. Self-compression in a pair of AR-coated plano-convex and plano-concave CaF_2 lenses (L1 and L2 in Supplementary Fig.7a) with the focal distances of $F_1 = 200\ mm$ and $F_2 = -50\ mm$ respectively was realized. The distance between the lenses was set to 135 mm while adjusting light intensity on the lens L2 for optimal self-compression. This determines an effective focal distance of the pair of lenses to be $F_{eff} = -667\ mm$ which results in moderately diverging beam after the lens L2 and allows avoidance of following filamentation as it was in the case of converging beam, described in the main text of the paper.

As a result of propagation through the pair of lenses 94-fs pulses were self-compressed to 32 fs pulse duration as it is confirmed by SHG FROG measurements (Supplementary Figure 8b-e). In order to avoid spatial-temporal distortions during propagation the output surface of the lens L2 vas reimaged with reduction on the SHG crystal of FROG apparatus.

Note that the shape of the lenses originate in a gradient of the thickness of the material across the beam: for the positive lens L1 $d_1^{center} = 2.9\ mm$, $d_1^{edge} = 2\ mm$, while for the negative lens L2 $d_2^{center} = 4\ mm$, $d_2^{edge} = 6.5\ mm$. in the case of negative lens the gradient results in thicker material for lower intensity and other way around, which potentially can improve the homogeneity of the self-compression across the beam.

Wavelength scalability of self-compression. By performing full 3D simulations, we have examined wavelength scalability of the self-compression process by means of determining spectral region in which lengths scales of nonlinear self-compression and spatial beam collapse can be separated. The simulations were performed for the case of self-compression in YAG. During the

simulations we kept the intensity of light incident to the YAG plate constant (at the level of 4.5 TW/cm^2), while the material thickness was varied in order to achieve optimal self-compression. Results of the simulations are presented in the supplementary figure 10 and reveal that the scales of self-compression and self-action/modulation instability/multiple filamentation are well separated in the spectral range 4.7 μm - 2.5 μm: (smooth temporal pulse shape and spatial beam profile indicate an absence of modulation instability). In fact, in YAG one can get even shorter pulses at shorter wavelengths (panel h vs panel g of the supplementary figure 10). The wavelength of 4.7 μm corresponds to the transparency edge of YAG. At shorter than 2.5 μm wavelengths fast spatial modulations and temporal pulse splitting take place. Additional observation which comes from the supplementary figure 10 is related to the material thickness: in order to achieve an optimum self-compression a thicker material is needed for shorter wavelength which mostly is determined by smaller value of GVD at shorter wavelength.

In Fig.11 calculated wavelength dependencies of some parameters characterizing the self-compression process are presented. The wavelength range of filamentation-assisted pulse compression is limited from the long-wavelength side by group-velocity dispersion (GVD), which grows in its absolute value (suppl. Fig.11a), lowering the soliton number of the pulse (suppl. Fig.11d), thus reducing the efficiency of pulse self-compression. On the short-wavelength side, when the absolute value of the GVD becomes too small (for wavelengths shorter than 3 μm), the self-compression length l_c (suppl. Fig.11e) becomes longer than the characteristic length l_m of modulation instabilities (MIs) (suppl. Fig.11b), which can be defined for practical purposes as the length within which the gain of noise in the angular spectrum reaches 100. To avoid beam breakup into multiple filaments as a result of MIs, the propagation length has to be limited to l_m, which is in this regime less than l_c, preventing pulse compression to minimum pulse widths. In suppl. Fig.11c the width of the self-compressing pulse is plotted as a function of the propagation path chosen as $z = \min\{l_c, l_m\}$. This dependence shows that filamentation-assisted pulse compression in YAG works best from approximately 3 to 4.5 μm.

The wavelength dependence of the pulse self-compression length l_c, i.e., the length at which the pulse of the self-compressing pulse reaches its minimum, is shown in suppl. Fig.11e. However, when the pulse self-compression length l_c becomes longer than the characteristic length of MI buildup l_m, the propagation length should be limited to l_m rather than l_c in order to avoid beam breakup into multiple filaments.

Calculations presented in supplementary figures 10 and 11 show that YAG is very well suited as material for filamentation-assisted self-compression of high-peak-power pulses with a central wavelength around 4 μm. While we cannot claim that YAG provides a global optimum among all the available material, it is certainly a very good choice among all the easily available materials. In particular GaAs and similar semiconductors are not suitable because they have (i) normal dispersion at around 4 μm and (ii) much lower laser damage thresholds. Diamond is also normally dispersive at 4 μm, while crystalline quartz and silica are absorptive in this wavelength range. CaF_2, MgF_2, and BaF_2 could potentially be useful, but a reasonable, and quite fragile,

compromise between spatial beam instabilities and self-compression, similar to that explained above for YAG, still needs to be found.

Energy scalability of self-compression. With the present OPCPA system generating 25-mJ level sub-100 fs pulses achievable by self-compression peak powers are below 1 TW level. On the other hand, scaling of the energy of mid-IR pulses towards 100-mJ level is a matter of scaling up the pump pulse energy, which can be done by building an additional amplification stage of picosecond pump. Flash lamp pumped Nd:YAG amplifiers based on 17-mm diameter Nd:YAG rods and capable of amplifying 100-ps pulses to the level of >1 J are commercially available. According to our estimates, an additional OPCPA stage pumped by 1-J pulses would allow us to generate >100 mJ pulses, which, by taking into account 40% compressor losses, would result in >60 mJ sub-100 fs pulses Our numerical simulations, presented in the supplementary figure 12 evidently show that provided our system generates 43 mJ or 160 mJ , 94-fs pulses, peak powers of respectively 1.3 TW and 5 TW can be achieved through a self-compression in 2-mm thick YAG plate. The simulations were performed by fixing an input field intensity at the level of 4.5 TW/cm^2.

Supplementary references

1 Kartashov, D. *et al.* Mid-infrared laser filamentation in molecular gases. *Opt. Lett.* **38**, 3194-3197 (2013).

2 http://www.fastlite.com/en/cat528877--Mozza.html.
